# The Multi-dimensional Mechanisms and Transformation Prospects of the Intratumoral Microbiota-Arginine Metabolism Axis in Tumor Progression and Immune Regulation

**DOI:** 10.7150/ijms.128450

**Published:** 2026-03-17

**Authors:** Shuyang Yu, Jinhua Chen, Wan Shu, Guanxiao Chen, Xiaoyu Shen, Shuangshuang Cheng, Kejun Dong, Hongbo Wang

**Affiliations:** 1 Department of Obstetrics and Gynecology, Union Hospital, Tongji Medical College, Huazhong University of Science and Technology, Wuhan, 430030, China.; 2 Clinical Research Center of Cancer Immunotherapy, Hubei, 430022, Wuhan, China.

**Keywords:** Intratumoral microbiota, Arginine, Metabolism, Tumor microenvironment, Immunity, Transformation

## Abstract

The intratumoral microbiota, as an important component of the tumor microenvironment (TME), impact tumor progression by regulating the arginine-ornithine metabolic axis. It has become a new frontier in tumor research. Arginine is a crucial amino acid in TME, and its metabolites, ornithine and polyamines, directly promote tumor proliferation and induce immunosuppression.

Intratumoral microbiota mainly exert their effects through two direct pathways: 1) arginine depletion, such as *Streptococcus* in gastric cancer. Specific intratumoral microbiota highly express arginine deiminase (ADI) or arginase (Arg) to consume arginine in the TME, leading to T cell dysfunction and enhancing immunosuppressive cells. 2) Ornithine/polyamines supplement, such as *fusobacteria* in esophageal cancer produce putrescine. The microbiota converts arginine into ornithine, which is then synthesized into polyamines, directly stimulating tumor cell proliferation and reshaping the immunosuppressive TME. Additionally, the metabolic products from the microbiota like short-chain fatty acids (SCFAs) and indole substances, can amplify these effects through signaling pathways including G protein-coupled receptor 43 (GPR43) and aryl hydrocarbon receptors (AHR).

The regulation of intratumoral microbiota-arginine metabolism axis has a “double-edged sword” characteristic, relying on the metabolic dependence of the different tumors, which provides a basis for precise treatment. Furthermore, strategies targeting the axis present great potential, including Arg1 inhibitors (CB-1158) in combination with immunotherapy, engineered probiotics to supply arginine and inhibit polyamine synthesis *in situ* within the TME. These advancements also indicate there is enormous progress from exploring the intratumoral microbiota-metabolism interaction to developing novel tumor microecological therapies.

## 1. Introduction

Metabolic reprogramming and immune regulation within the tumor microenvironment (TME) are significant driving factors for tumor occurrence and development, and the intratumoral microbiota were discovered as a potential sight to this field in recent years 1. Traditional biology holds that tumors create a sterile environment, however, recent high-throughput sequencing and spatial imaging reshaped this view 2. The distribution of the intratumoral microbiota exhibits significant heterogeneity, and its enrichment is positively correlated with the location and exposure to the external environment 2. Cancers exposed to outside present the more abundant and diverse intratumoral microbiota, such as melanoma, colorectal cancer (CRC), and lung cancer 2-5. Nevertheless, increasing evidence indicated that certain microbiota also exists in internal tumors including those in the liver, pancreas and breast 6-8. These microbiota may enter the TME through hematogenous or lymphatic metastasis with the damaged barrier. Also, there are low-biomass microbial DNA and lipopolysaccharide (LPS) signals within brain tumors protected by the blood-brain barrier like glioblastoma (GBM) 9, 10, which suggest that intratumoral microbiota is far more extensive than previously thought. These intratumoral microbiota further profoundly affect the nutrient supply and signal transduction in the TME through metabolism and secreted products, thereby influencing immune surveillance and tumor progression 11, 12.

Arginine serves as a joint among protein metabolism, polyamine production, nitric oxide (NO) synthesis and the urea cycle, determining the cellular proliferation and function 13. The arginine-ornithine metabolic pathway as a crucial link connects the functions of immune cells and the proliferation of tumor cells 13-15. Importantly, although T cells have the capacity for arginine synthesis, their metabolic demand exceeds synthesis in the activated state, hence exogenous uptake is necessary for proliferation and effector functions. Also, ornithine and its derivatives polyamines act as important regulatory factors for cell proliferation and differentiation 13, 15, 16. Due to its characteristics in metabolism and immune, arginine is crucial for microbial regulation compared to other natural amino acids. Tumor cells often upregulate arginine metabolic related enzymes including arginase (Arg) and ornithine decarboxylase (ODC), to serve for their rapid growth with inducing immunosuppression. For example, the immunosuppressive tumor-associated macrophages (TAM) highly express ARG1 and consume arginine 13, 17. It is worth noting that the intratumoral microbiota also participates in arginine and ornithine metabolic regulation 18. Numerous studies have shown that certain intratumoral bacteria can efficiently decompose arginine or synthesize ornithine and polyamine metabolites, thereby locally construct a TAM favorable for the tumor, which inhibit anti-tumor immunity and promote tumor growth 18, 19.

**Figure 1:**
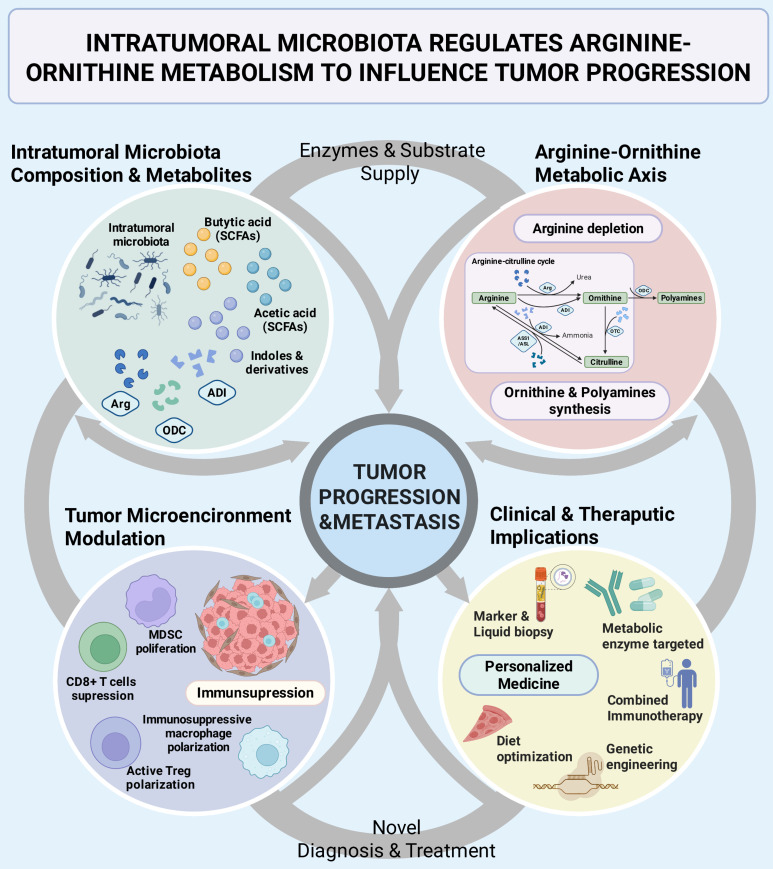
** Intertumoral microbiota regulates arginine-ornithine metabolism to influence tumor progression.** The interaction between intratumoral microbiota and the arginine-ornithine metabolic axis was systematically clarified, which jointly impact tumor progression and the tumor microenvironment, as well as provide inspiration for clinical applications. Intratumoral microbiota and their metabolites regulate tumor progression and the immune microenvironment within the tumor through processes such as arginine depletion and polyamine production. The microbiota-metabolism-immune axis in tumor also provides novel perspectives for clinical diagnosis and treatment. SCFAs, short chain fatty acids; Arg, arginase; ADI, arginine deiminase; ODC, ornithine decarboxylase; OTC, ornithine transcarbamylase; ASS1, argininosuccinate synthetase; ASL, argininosuccinatelyase; MDSC, myeloid-derived suppressor cell.

This review will focus on how does the intratumoral microbiota impact tumor progression by regulating the arginine-ornithine metabolism, to systematically summarize the progress of its mechanisms and explore its clinical translations (Figure 1). We will mainly discuss: 1) the effects of microbial arginine metabolic enzyme on immunity and metabolism in the TME; 2) the role of microbiota-derived ornithine metabolism in tumor proliferation and metastasis; 3) the balance of the ornithine-citrulline cycle in tumor metabolism and homeostasis; 4) the indirect regulation by microbial metabolites to related signaling pathways and overall metabolic reprogramming mechanisms; 5) some discoveries in typical cancers (Figure 1). Finally, we will look forward to the new biomarkers and intervention strategies based on the intratumoral microbiota-metabolism axis, including probiotic engineering, metabolic enzyme inhibitors, combined immunotherapy, and other creative progressions (Figure 1).

## 2. Metabolic regulation of arginine and ornithine through the microbiota

### 2.1. Arginine degradation and metabolic competition mediated by intratumoral microbiota

The metabolic state of arginine in the TME determines the function of immune cells and tumor cells. Some intratumoral microbiota can highly express arginine metabolic enzymes to consume arginine, causing local arginine deficiency and resulting in immunosuppressive effects 18, 20.

**Figure 2:**
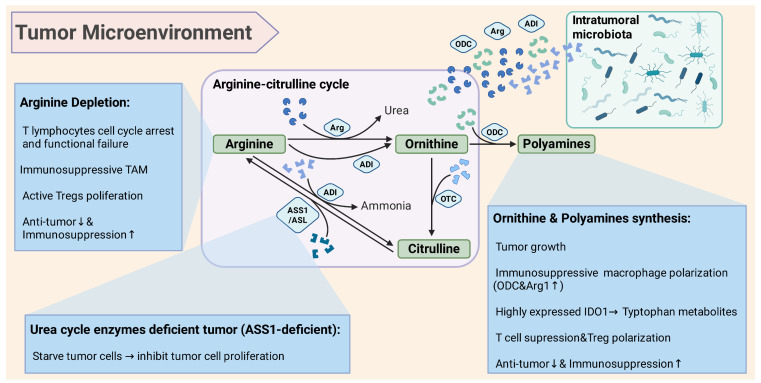
** Intratumoral microbiota affects the immune microenvironment and tumor progression through arginine and ornithine metabolism.** Arginine depletion: Intratumoral microbiota highly expressed ADI or Arg, consume arginine in the TME to induce the failure of T cell function and enhance immunosuppressive cells (Treg). Ornithine & polyamine supply: Intratumoral microbiota metabolizes arginine into ornithine, then synthesize polyamines from ornithine to stimulate tumor cell proliferation and reshape the immunosuppressive TME. A positive feedback loop forms to promote tumor deterioration through the ornithine-polyamine axis. Ornithine-citrulline cycle: As part of the urea cycle, arginine can be hydrolyzed by Arg to generate ornithine, which is combined with OTC to form citrulline. Citrulline then forms arginine under the action of ASS1/ASL. However, in tumors with arginine synthesis defects (ASS1 deficiency), arginine depletion can starve tumor cells to limit tumor progression. Intratumoral microbiota regulates the direction and degree of arginine conversion to ornithine or citrulline, altering the dynamic of the metabolic cycle and thereby affect cell proliferation and TME homeostasis. Arg, arginase; ADI, arginine deiminase; ODC, ornithine decarboxylase; OTC, ornithine transcarbamylase; ASS1, argininosuccinate synthetase; ASL, argininosuccinatelyase; Tregs, regulatory T cells; IDO1, indoleamine 2,3-dioxygenase 1; TAM, tumor-associated macrophages.

On one hand, bacterial arginine deiminase (ADI) can hydrolyze arginine into ornithine, directly depleting the tumor arginine supply 18 (Figure 2). For instance, highly abundant *Staphylococcus anginosus* (*S. anginosus*) colonized in gastric cancer was found to have an active arginine metabolism, which can rapidly consume arginine to generate ornithine, leading to a significant decreased arginine in co-cultured tumor models 18. Further compared with sterile controls, *S. anginosus* colonization could reduce arginine levels with increased ornithine in tumor tissues, and accelerate tumor growth *in vivo* 18. The microbiota-mediated arginine depletion also has a profound impact on immune cells. The proliferation and effector functions of T lymphocytes depend on a sufficient supply of arginine, and T cells will enter cell cycle arrest and functional failure under deficient arginine 18, 21. The reduction of the competing substrates for inducible nitric oxide synthase (iNOS) in macrophages decreases the NO-mediated anti-tumor killing. At the same time, arginine depletion induces an increased tendency toward regulatory immune cells (Figure 2). Studies found that certain *Proteobacteria* can enhance the inhibitory function of regulatory T cells (Treg) in the TME by consuming host arginine, then weaken the anti-tumor immune response 22, 23. Lacking these arginine-consuming bacteria increased the serum arginine levels in mice, which promote the excessive activation of mTOR signaling in Tregs with altered functional state 22. Conversely, when there were abundant arginine-consuming bacteria, arginine levels decreased and the mTOR pathway was restricted in T cells, that is more conducive to form Tregs mediated immune tolerance 22, 23.

On the other hand, bacterial Arg can also participate in arginine degradation. Some pathogenic bacteria like *Helicobacter pylori* (*H. pylori*) expressed Arg can directly compete with host iNOS for substrates, decomposing arginine into ornithine and urea 24. The arginine metabolism of *H. pylori* has been proven to significantly inhibit macrophages from synthesizing NO, thus promoting bacteria and tumors to evade immune clearance 24, 25. Similarly, other Arg expressing microbiota enriched in tumor tissues may also play a similar role in that arginine depletion suppresses the effective function of immune cells, and the produced ornithine may be further utilized by tumor cells 18, 26 (Figure 2).

Moreover, arginine itself is an essential nutrient for tumors that cannot synthesize arginine due to the deficiency of urea cycle enzymes (Figure 2). Therefore, microbiota-mediated arginine depletion may directly starve some tumor cells 16, 27. One of the therapies for tumors with arginine synthesis defects in clinical settings is to inject PEGylated arginine deiminase (ADI-PEG20) from microbiota to rapidly clear arginine and starve tumor cells, such as argininosuccinate synthetase (ASS1)-deficient liver cancer and melanoma 28, 29 (Figure 2). However, the regulation of arginine metabolism by the microbiota may vary depending on the various TME, leading to complex immune and metabolic regulation. Studies found that after colonizing a strain of bacteria with defective arginine metabolism to reduce the microbial breakdown of arginine, it actually accelerated tumor progression in a mouse model of CRC 26. The excessive arginine accumulation was utilized by tumors and immunosuppressive cells, further activating the NO synthesis and polyamine synthesis. Thus, it promotes angiogenesis and immunosuppressive macrophage polarization, and activates the proliferative Wnt/β-catenin signaling, ultimately accelerating tumor progression 23, 26.

Overall, in most immunogenic tumors, the local arginine depletion by the intratumoral microbiota often favors tumor escape from immunity, by inhibiting T cells, NK cells and so on 29, 37 (Figure 2). While in some arginine-dependent tumors, the arginine depletion by microbiota may inhibit tumor cell proliferation (Figure 2). Conversely, if the microbiota increases the local arginine supply, it may both supply the tumor and stimulate immunosuppressive pathways, such as the Arg pathway of immunosuppressive macrophages, finally manifesting a tumor-promoting effect 17.

### 2.2. The synthesis of ornithine and polyamines mediated by intratumoral microbiota

Ornithine is the direct product of arginine degradation, and its accumulation in the TME has various effects on tumor progression. Firstly, ornithine can be converted into polyamines including putrescine, spermidine, and spermine under the action of ODC, which are important promoters of cell proliferation and differentiation 30 (Figure 2). Polyamines can facilitate nucleic acid and protein synthesis, stabilize nucleosome, and affect the cell cycle by regulating the modification of translation initiation factor EIF5A 31. Furthermore, polyamines not only promote tumor growth and invasion, but can also form an immunosuppressive environment through the reprogramming and feedback of immune cells (Figure 2). Spermine can induce high expressed indoleamine 2,3-dioxygenase (IDO1) in tumor cells and DCs, which lead to the accumulation of tryptophan metabolites, further inhibiting T cell and driving the differentiation of Tregs 32, 33. For instance, high levels of spermine can induce dysfunction of dendritic cells (DCs) and inhibit T cell proliferation, thereby help tumors evade immune surveillance 31, 34. The level of polyamines within tumor is related to the malignancy and poor prognosis of tumors, and macrophages with high expressed ODC and Arg1 can support tumor growth and inhibit anti-tumor immunity by generating polyamines 20, 31 (Figure 2).

Recent studies proved that *F. nucleatum* is a common symbiont in tumors like CRC, which is also highly enriched in esophageal squamous cell carcinoma (ESCC) 19. Clinical study showed that the *F. nucleatum* in ESCC tissues was significantly negatively correlated with the prognosis, patients with high abundance had a shorter survival period 19, 35. *F. nucleatum* could invade ESCC epithelial cells and generated high levels of polyamine like putrescine, which disrupted the polyamine metabolic balance and promoted the malignant proliferation of tumor cells 35, 36. This verified the crucial role of the ODC-polyamine metabolism from intratumoral microbiota in tumor progression. Furthermore, *F. nucleatum* is associated with enhanced tumor progression and metastasis in CRC, and its produced polyamines and toxic metabolites may promote tumor occurrence through activating Wnt/β-catenin signaling 35, 37, 38. Besides *F. nucleatum*, others such as *S. anginosus* and *Escherichia coli* (*E. coli*) may also contribute to polyamine metabolic activity. *S. anginosus* increases ornithine production from arginine, accompanied by the massive accumulation of downstream polyamine derivatives such as N¹-acetylseramine and N⁸-acetylornithine in gastric tumor 18. Additionally exogenous supplementation of arginine and its downstream acyl spermidine significantly accelerated tumor growth in gastric cancer xenograft model 21, 39. Therefore, it can be inferred that *S. anginosus* produces ornithine and polyamines through arginine metabolism, and activates the polyamine pathway to promote tumor proliferation.

In addition to immune and proliferation regulation, the microbiota-arginine-polyamine axis also plays a crucial role in tumor blood and lymphatic vessels. As a downstream of the microbial arginine metabolism, polyamines are necessary regulators in the vascular characteristic. Recent studies showed that, the increased polyamine in the TME from intratumoral microbiota such as *F. nucleatum* significantly upregulated the expression of vascular endothelial growth factor (VEGF) and basic fibroblast growth factor (bFGF), through the hypusination of EIF5A, thereby accelerating pathological angiogenesis 19, 40. Moreover, the microbial regulation of arginine directly affects the level of NO in the TME. In CRC models, it has been demonstrated that arginine catabolism mediated by certain microbiota can drive the angiogenesis by activating the Wnt/β-catenin signaling pathway in endothelial cells, promoting vessel branching and damaging vascular integrity, thereby facilitating hematogenous metastasis 26. It is noteworthy that this metabolic axis also impacts the lymphatic system. Microbiota derived ornithine can be utilized by TAMs to drive polyamine-dependent metabolic reprogramming, which further induce a lymphangiogenic phenotype characterized by high secretion of VEGF-C and VEGF-D 41, 42. This process not only expands the lymphatic network within the tumor, but also inhibits the recruitment of effector T cells to the lymphatic vessels, thereby protecting metastasis 43. Therefore, the intratumoral microbiota regulates the TME structure by balancing the conversion of arginine to polyamine, suggesting that targeting this axis may have dual benefits of blocking nutrient supply and its lymph escape pathways.

### 2.3. Arginine-citrulline cycle: the influence of microbiota on tumor metabolism and homeostasis

Within the arginine metabolism, ornithine and citrulline can be interconverted through the urea cycle and related branches, forming an ornithine-citrulline cycle 44, 45 (Figure 2). This cycle is crucial for maintaining the metabolism and homeostasis of TME, while intratumoral microbiota further increases the dynamics of this cycle. As a part of the urea cycle, arginine can be hydrolyzed to generate ornithine by Arg. And ornithine is combined with carbamoyl phosphate by ornithine transcarbamylase (OTC) to form citrulline, which then integrates with aspartate to generate arginine under the action of ASS1/ argininosuccinatelyase (ASL) 16, 45 (Figure 2).

However, ASS1/ASL lacking tumor cannot effectively utilize citrulline to regenerate arginine, hence exogenous arginine becomes a limiting factor for tumor growth 46, 47. The arginine metabolism of microbiota can disturb the balance of this cycle (Figure 2). When the intratumoral bacteria highly express ADI, arginine is largely converted into citrulline and ammonia that is not utilized under ASS1 deficiency 47. Conversely, if the microbiota mainly consumes arginine through Arg pathway, the produced ornithine may be utilized by tumor cells to synthesize polyamines or other molecules, thereby partially supplying for tumor cells 13, 16, 30 (Figure 2). PEG-arginase drugs can convert arginine to ornithine, but the potential risk is that excessive ornithine may synthesize polyamines with tumor promoting effects 48, 49. Thus, the microbiota mediated transformation of arginine to ornithine or citrulline has a dual impact on the tumor metabolism, depending on the ability of ornithine/citrulline utilization and the activity of downstream pathways of the tumor (Figure 2). If the tumor can effectively utilize ornithine to synthesize polyamines, the microbial Arg will support it. If the tumor cannot utilize citrulline and is arginine-dependent, the ADI pathway will cause arginine starvation to damage it.

Additionally, the by-products of arginine metabolism including ammonia and urea, also impact the metabolic and immune TME. High levels of ammonia generated by microbial ADI can increase the local pH and change the acidic state of the TME, which have been identified as a major driver of T cell dysfunction 32, 50, 51. A recent study demonstrated that ammonia induced metabolic reprogramming in tumor-infiltrating T cells with lysosomal and mitochondrial stress, ultimately enhancing T cell exhaustion 52. Tumor cells also recycle ammonia as a fundamental nitrogen source via glutamate dehydrogenase (GDH), to support amino acid synthesis and metabolism, thereby exhibiting a survival advantage under nutrient-deprived conditions 53. In addition, the accumulation of urea from enhanced Arg further shapes the immunosuppressive TME. Urea cycle dysregulation is recognized not only as a metabolic hallmark of cancer also as a mechanism to evade immune surveillance. It promotes the recruitment and function of myeloid-derived suppressor cells (MDSCs), which further deplete local arginine and secrete inhibitory cytokines, resulting in positive feedback of immune exclusion 54. Furthermore, these by-products may influence the efficacy of chemotherapy and immunotherapy, through modulate the local pH in TME 55.

Moreover, the correlative pathways also make significant contributions to tumor progression and immune regulation in the presence of arginine regulated microbiota. Ornithine produced through the Arg pathway can be further converted into proline by ornithine aminotransferase, which promotes collagen synthesis and tumor fibrosis 56-58. In highly fibrotic tumors such as PDAC, studies found that the enriched *Pseudomonas* present massive Arg and proline metabolic genes. The microbial-driven arginine-proline axis enhance tumor matrix fibrosis and physical barrier for immune cell infiltration by promoting collagen synthesis. It also supports the stress survival of cancer cells as a key bypass for polyamine biosynthesis 16, 59. Recent spatial multi-omics study further confirmed that this microbial-metabolism interaction is important for the malignant progression and immune evasion of PDAC 60, 61. Similarly, aspartate is used to rescue arginine synthesis through the ASS1 pathway under the arginine deficiency, further result in the aspartate deficiency. Since aspartate is an important nitrogen source for nucleotide biosynthesis and DNA repair, excessive microbial-induced aspartate depletion limits tumor cell proliferation and increases their sensitivity to oxidative stress 62. This microbial-driven metabolic redirection provides new logical support for starvation therapy targeting ASS1-deficient tumors. Although there are relatively few studies about it, intratumoral microbial-driven arginine reprogramming indirectly affects other amino acid biosynthesis including proline and aspartate 16, 59. It also represents an innovative frontier: targeting microbial arginine metabolism within tumor can simultaneously disrupt multiple interdependent amino acid pathways, providing synergistic treatment for tumors such as PDAC.

### 2.4. The indirect regulation of the arginine pathway by microbial metabolites

In addition to directly participating in arginine and ornithine metabolism, other metabolites and signaling molecules produced by intratumoral microbiota can also affect the arginine metabolism and immune metabolic responses of the host 63. The most typical include short-chain fatty acids (SCFAs) and derivatives of tryptophan metabolism 64-66.

#### 2.4.1. Short-chain fatty acids (SCFAs)

SCFAs as the fermentation products of the microbiota, are widely present in the intestinal tract and TME. They can regulate the metabolism of immune cells through G protein-coupled receptors (GPCRs) and epigenetic pathways 67-69. SCFAs produced by intratumoral microbiota, including acetic acid from *Bifidobacterium* and butyric acid from *Lactobacillus*, may also be circulated from the intestinal microbiota to TME 70-72.

**Figure 3:**
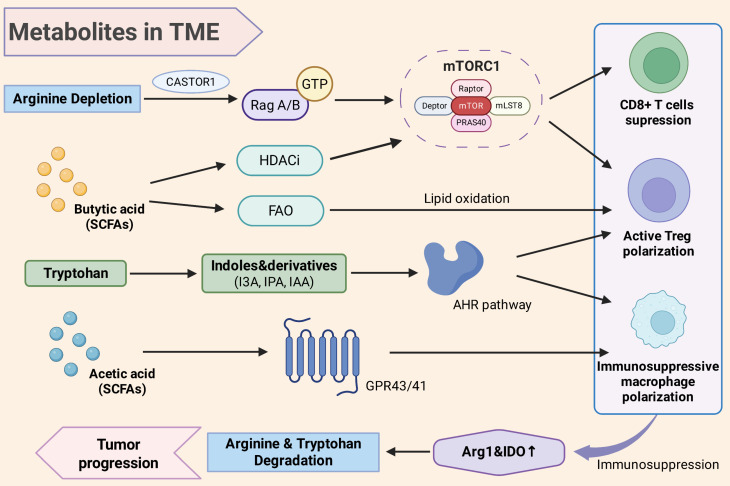
** The metabolites of intratumoral microbiota impact TME through signaling pathways.** Arginine can activate Rag GTPase through CASTOR1, thereby promoting the activation of mTORC1. Under arginine depletion induced by intratumoral microbiota, the mTOR signal in T cells weakens, inducing Treg differentiation rather than effector T cells. Butyric acid can regulate the mTORC1 signal through its HDACi activity, and promote the metabolic bias towards fatty acid oxidation in regulatory T cells with enhanced the differentiation. Acetic acid in SCFAs acts on macrophages through GPR43/41, promoting their immunosuppressive transformation. Tryptophan produces indole and its derivatives (I3A, IPA, IAA) to activate the AHR signaling pathways, which promotes immunosuppressive macrophages and the differentiation of Treg cells, and inhibits the proliferation of effector T cells with an immunotolerant TME. The metabolite-created immunotolerant TME is accompanied by the activation of Arg1 in macrophages and IDO in DC, further accelerate the arginine depletion and tryptophan, finally strengthening immunosuppression and promoting tumor progression. TME, tumor microenvironment; SCFAs, short chain fatty acids; Arg, arginase; HDACi, histone deacetylase inhibitor; FAO, fatty acid oxidation; I3A, indole-3-carboxaldehyde; IPA, indole propionic acid; IAA, indole-3-acetic acid; AHR, aryl hydrocarbon receptor; GPR43/41, G protein-coupled receptor 43/41; Treg, regulatory T cell; IDO, indoleamine 2,3-dioxygenase.

SCFAs impact the arginine metabolism by altering the immune cell functions, thereby regulating the enzymes activity including iNOS and Arg1^64^, ^73^, ^74^ (Figure 3). Butyric acid with histone deacetylase inhibitor (HDACi) activity, can promote the fatty acid oxidation in Tregs and enhance their differentiation 75, 76 (Figure 3). The activated Tregs is accompanied by the activated immune-inhibiting metabolic pathway Arg1 and IDO in macrophages and DCs, which force the competitive metabolism of arginine and tryptophan with effector T cells inhibition 22, 77. And acetic acid acts on its receptor GPR43/41 of macrophages, and its high concentrations can inhibit glycolysis and pro-inflammation in macrophages, driving its immunosuppressive transformation 78, 79 (Figure 3). Immunosuppressive macrophages typically express higher Arg1 and further secrete arginine-derived polyamines with the reinforced immunosuppressive metabolic effect 17, 20 (Figure 3). *Bifidobacterium* colonized in CRC can secrete metabolites including lactic acid and acetic acid, which facilitate tumor growth and immune escape, leading to poor patient prognosis 80. Therefore, SCFAs promote pro-inflammatory and immunosuppressive phenotypes, further upregulate the Arg1 activity and arginine consumption with polyamine production in the TME, finally contribute to the tumor immune escape.

Moreover, SCFAs as metabolic substrates can participate in the tricarboxylic acid cycle of T cells and NK cells, in order to provide energy and regulate their differentiation 81 (Figure 3). And moderate exposure of SCFAs can also perform anti-tumor immunity. Butyric acid can inhibit HDAC and enhance mTOR signaling, to improve the glucose metabolism and cytotoxic activity of CD8+ T cells, which may counteract the adverse effects of arginine deficiency 81. In summary, SCFAs produced by intratumoral microbiota are important regulators of the immune metabolic reprogramming in the TME, which can both enhance anti-tumor immune and induce immune tolerance, depending on the dose, cell type, and time window 81, 82.

#### 2.4.2. Metabolic derivatives of tryptophan

The tryptophan metabolites and aryl hydrocarbon receptors (AHR) pathways, are also key components of the microbiota-host immune metabolic axis 61, 83, 84. Many intestinal and intratumoral microbiota can metabolize tryptophan to produce indoles and their derivatives, like indole-3-carboxaldehyde (I3A), indole-3-propionic acid (IPA) and indole-3-acetic acid (IAA). 61, 85. These microbiota-derived indole compounds as ligands of the AHR, can activate the AHR signaling pathway in immune cells 86 (Figure 3). AHR is a ligand-dependent transcription factor expressed in various immune cells. Its activation drives the transcription of downstream genes, thereby influencing the differentiation and metabolic state of immune cells 86. In the TME, the continuous activation of AHR signals is often associated with immunosuppression 61, 86, 87. Studies found that TAMs in PDAC exhibit high AHR activity, which might be concerned with the continuous exposure to indole metabolites from microbiota 61. Macrophages with high AHR tend to secrete immunosuppressive factors with expressing Arg1, thereby creating an immunotolerant TME 61 (Figure 3). Additionally, the activated AHR can promote the differentiation and function of Treg cells and inhibit the effective T cells 88, 89 (Figure 3). Blocking AHR signals can suppress tumor growth and enhance anti-tumor immune responses in tumor models, supporting the crucial role of AHR in immunosuppressive metabolism 90. Since AHR is closely related to the IDO-tryptophan metabolism, the AHR activated by intratumoral microbiota often occurs under co-dysregulation of arginine and tryptophan metabolism, together with highly expressed Arg1 and IDO in an immunosuppressive TME 33, 61, 83 (Figure 3). This means that the activated AHR may synergize with bacterial Arg1 pathway to accelerate the depletion of arginine and tryptophan, causing immune tolerance through downstream signaling.

#### 2.4.3. mTOR signaling pathway

Furthermore, the mTOR signaling pathway acts as the cellular central processor to sense amino acid abundance and regulate metabolism 91-93. Arginine is an important upstream stimulus for mTORC1 complex, and the sense of the arginine concentration mainly relies on CASTOR family proteins, which contains CASTOR1 and CASTOR2. CASTOR1 is the core sensor that directly binds to arginine. When arginine is deficient, CASTOR1 binds to the GATOR2 complex and inhibits its activity, thereby blocking the activation of mTORC1. Therefore, CASTOR1 has a specific arginine binding site, serving as a trigger for concentration sensing. While CASTOR2 as a structural stabilizer form a heterodimer with CASTOR1 to adjusting the sensing sensitivity by influencing the conformation of CASTOR1. Sufficient arginine can activate Rag GTPase through amino acid sensors CASTOR1, then promote mTORC1 localized on the lysosomal membrane and activated by Rheb 94, 95. The activated mTORC1 further forces anabolic metabolism, inhibits autophagy, and supports cell growth and proliferation 96, 97.

Intratumoral microbiota also affects the activity of the mTOR pathway in immune cells and tumor cells by controlling arginine levels (Figure 3). It has been verified that when the microbiota extensively decomposes arginine, mTOR signal in T cells is decreased, which prefer to inducing Treg differentiation rather than effective T cells 22. Conversely, if probiotics metabolism increases the arginine level, the enhanced mTOR activity may drive its differentiation towards the effective helper T cell/cytotoxic T lymphocyte (Th1/CTL), improving anti-tumor ability 1, 22. Therefore, engineered strains were used to provide arginine locally, its results confirmed a significant increase in tumor-infiltrating T cells with enhanced expression of downstream effector molecules like IFN-γ, ultimately enhancing the immunotherapy 98, 99. Moreover, SCFAs can reprogram T cell metabolism by affecting the AMPK and Akt-mTOR axis 82, 100, 101 (Figure 3).

Moreover, tumor cells also downregulate anabolic metabolism through mTORC1 pathway when faced with arginine starvation from intratumoral microbiota. However, unlike immune cells, tumor cells exhibit a strong metabolic flexibility 94, 102. They perform a stronger autophagy adaptation ability to supply arginine by degrading intracellular proteins. Tumor cells also compensatorily activate the mTORC2 pathway through lysosomal localization. mTORC2 as a key upstream of the Akt pathway, its enhanced activity can maintain the phosphorylation of Akt in arginine-deficient environments, thereby activating autophagy to recycle endogenous arginine and regulating the anti-apoptotic ability of tumor cells 102. Therefore, this explains why some tumors can still survive under severe arginine deprivation, by enhancing invasiveness to achieve metabolic escape.

## 3. Microbiota-arginine/ornithine metabolic axis in typical cancers

Due to the various locations, microenvironments and microbiota compositions among different cancers, the mechanisms by which the intratumoral microbiota affect metabolism in TME also differ. Here, we will analyze and summarize the research progress of the microbiota-arginine/ornithine axis combining with representative cancers:

### 3.1. Colorectal cancer (CRC)

CRC is the tumor with the most extensive research on microbiota that both inside and outside the tumor may be involved in tumor progression. *F. nucleatum* is a recognized intratumoral bacterium in CRC, and its abundance is significantly correlated with poor prognosis and increased liver metastasis 103-105. Besides adhering to epithelial cells and activating oncogenic signals, *F. nucleatum* also impact the TME through metabolism 35, 106. Its produced polyamines may promote tumor cell proliferation and inhibit immune surveillance in TME, through Wnt/β-catenin pathway activation and IL-10 production from myeloid cells 107-109. *E. coli* is also a frequently detected intratumoral strain in CRC 110, 111. Some of strains carry the pks virulence inducing DNA damage, while others are involved in metabolic regulation. Studies showed that the arginine degradation by intratumoral microbiota is significantly enhanced in CRC, especially some *E. coli* can consume arginine largely through the arginine succinyltransferase (AST) pathway 26, 111. In mouse models, inhibiting microbial arginine metabolism could increase arginine levels in tumors, but induced a series of tumor-promoting changes, including increased NO and polyamine synthesis, enhanced angiogenesis, increased infiltration of immunosuppressive macrophages, and upregulation of Wnt signaling, ultimately promoting tumor progression 26. It suggested that the arginine metabolism of intratumoral microbiota may limit tumor growth, also weaken immunity. Clinically, CRC patients attempted to take high-dose arginine orally to improve immune efficacy, but the effect was not obvious, indicating that the metabolic regulation of host microbiota-arginine is extremely complex 98, 112. Given the rich and easily modifiable microbiota, CRC is also an important field to exploring microbiota metabolic therapy, and its specific mechanism and intervention still need to be further explored.

### 3.2. Pancreatic cancer (PDAC)

The TME of PDAC presents highly fibrotic and immunosuppressive, and has been confirmed to contain rich intratumoral microbiota 60, 113. They mostly originate from the intestine or oral cavity, and enter the pancreas through the bloodstream or bile ducts, such as *Clostridiales*, *Actinomycetales*, and Gram-negative bacteria 114-116. Studies found that intratumoral microbiota imbalance can drive innate and adaptive immune suppression, including increased TAMs and MDSCs, and reduced the infiltration of effective T cells 113, 117. Pushalkar *et al.* verified that the tumor growth was slower in germ-free mice, and the microbiota removed by antibiotics could reactivate anti-tumor immunity and improve the efficacy of checkpoint inhibitors (ICB) 113, 118. In terms of metabolism, the intratumoral microbiota may utilize the abundant free amino acids to compete or cooperate with tumor cells 8, 61, 119. *Pseudomonas* in PDAC have enriched Arg enzyme and proline metabolic genes, which are speculated to consume arginine and produce metabolites to affect anti-tumor immunity in the TME 8. Additionally, there is evidence that the macrophages in PDAC performed high AHR activity and immunosuppressive phenotype, due to the long-term exposure to indole derivatives from microbiota metabolic products 61, 119. It demonstrated that the intratumoral microbiota strengthens the suppressive immune metabolism of Arg1 and IDO via the indole-AHR axis, synergistically enhancing tumor immune escape. Clinically, *H. pylori* DNA has also been detected within tumors of PDAC patients. It is supposed that the Arg activity of *H. pylori* may play a role to inhibit macrophages produced NO in the pancreatic TME 120, 121. In summary, the intratumoral microbiota exacerbates the "cold tumor" of PDAC through arginine metabolism, the interventions targeting intratumoral microbiota are expected to be explored and improve the immune microenvironment 122, 123.

### 3.3. Bladder cancer (BCa)

BCa has unique microbial characteristics in the urinary tract, which can regulate arginine metabolism in the TME. Sequencing studies revealed specific intratumoral microbiota different from urinary microbiota, like *F. nucleatum* 124. *F. nucleatum* is known to convert arginine into ornithine and polyamines, which directly promote tumor cell proliferation and shape an immunosuppressive TME. Besides, the microbiota expressing ADI or Arg locally consume arginine, thereby impairing the T cells proliferation and the NO synthesis by macrophages 22. The metabolic by-products of microbial arginine degradation further affect the immune TME. Excessive polyamines in BCa can suppress the entry of arginine into immune cells and NO-mediated tumor killing activity, then inhibiting anti-tumor immunity. In clinical study, the attenuated *Mycobacterium tuberculosis* stimulated a strong iNOS activity in the bladder, elevating local NO levels, thereby promoting the killing of tumor cells 125. Moreover, many invasive BCa also perform loss of ASS1 with arginine dependence. Therefore, ADI-PEG20 significantly inhibits the growth of ASS1-deficient BCa *in vitro* and in mouse models by depleting extracellular arginine 126. This highlights that the microbiota-arginine/ornithine metabolic axis is crucial in BCa progression and anti-tumor immune response. Targeting the axis by modulating the intratumoral microbiota and arginine metabolism is an innovative strategy to enhance anti-tumor immunity and drives new clinical research in BCa.

### 3.4. Melanoma

Melanoma is an immunogenic cancer, the arginine metabolism and microbiota in the TME significantly affect tumor immunity. Most melanomas lack ASS1, which cannot synthesize arginine, hence it can be a therapeutic point targeted by arginine-degrading enzymes 127. Clinical studies explored that ADI-PEG20 combined with chemotherapy showed persistent control activity in advanced ASS1-negative melanomas 28. Conversely, arginine supplement can enhance CD8⁺ T cell tumor infiltration and improve the efficacy of anti-PD-1 checkpoint therapy in mouse models 128. Furthermore, microbiota can systematically regulate arginine levels and immune cell function. In melanoma tissues, translocated bacteria from the oral cavity and skin are frequently detected, such as *Porphyromonas gingivalis* (*P. gingivalis*) and *F. nucleatum* 129. They carried lipopolysaccharides (LPS) and metabolites stimulate NF-κB through TLR2/4 signaling pathway, promoting the polarization of immunosuppressive macrophages with upregulated Arg1 expression 1,129. Research revealed that the intestinal probiotic *Limosilactobacillus reuteri* can migrate to the melanoma and utilize tryptophan to synthesize I3A. I3A then activates the AhR signaling pathway to induce an efficient state of CD8+ T cells, significantly enhancing the ICB efficacy 130. However, the high abundance bacteria with degrading arginine in TME may weaken this enhancing efficacy. Studies have found that the level of L-arginine in the circulation is an independent predictor of ICB efficacy in melanoma patients, and the abundance of arginine-degrading enzymes in intratumoral microbiota is negatively correlated with this 122, 131.

### 3.5. Mesothelioma

Malignant mesothelioma as an aggressive pleural tumor, is significantly influenced by arginine metabolism. One of the notable metabolic features is that about 50% tumors present absence of ASS1, resulting in absolutely dependence on exogenous arginine 132. In the Phase III clinical trial ATOMIC-Meso, researchers conducted a randomized, double-blind controlled study on 249 patients with non-epithelioid mesothelioma 133. Its results showed that adding ADI-PEG20 (peglotamycin) to the standard chemotherapy regimen (pemetrexed + cisplatin) significantly prolonged the overall survival (OS) and progression-free survival (PFS) of the patients, highlighting a new metabolic efficient therapy 133. The arginine/ornithine axis also reshapes the immune TME of mesothelioma. Mesothelioma triggers a chronic inflammatory TME dominated by TAM and neutrophils with high ARG1 and iNOS dysregulation, which converts arginine into ornithine and polyamines rather than anti-tumor NO 128. Excessive polyamines and ornithine enhance fibrosis and immunosuppression, promoting tumor growth. Although mesothelioma typically occurs in sterile sites, the systemic microbiota may affect its course through regulating host arginine and immune microbiota 134. Eliminating bacteria that consume arginine has been proven to increase arginine levels and enhance CD8⁺ T cell activity in distant tumors, which might be used to enhance anti-tumor immunity in mesothelioma patients 23, 135.

## 4. The potential for diagnosis and treatment

### 4.1. Biomarkers and prognosis

The characteristics of intratumoral microbiota and their metabolites have been regarded as potential diagnostic and prognostic markers recently 6, 18, 140-142. Studies on gastric cancer discovered that patients with high alpha diversity of intratumoral microbiota have better prognosis, while certain bacteria with excessive abundance are present in poor prognosis subtypes such as *S. anginosus* and *F. nucleatum* 18, 143-145 (Table 2). The classification according to composition of intratumoral microbiota can independently predict the survival of patients from the clinical stage 18. Additionally, the microbial signals of metabolic pathways can also serve as markers 11, 146. Patients with enriched *S. anginosus* in gastric cancer present an upregulated arginine metabolic pathway, which has been proposed as a risk factor 18. And the abundance of *F. nucleatum* in tumors and feces has been explored as an indicator for early screening and prognosis assessment of CRC 104, 147. High abundance indicates a more aggressive tumor and poorer prognosis, which may be partly attributed to its pro-tumor effects of polyamine metabolism 104 (Table 2).

Moreover, the expression of host immune metabolic enzymes also reflects the role of the microbiota. High level of serum Arg1 in head and neck squamous cell carcinoma (HNSCC) patients is associated with poor prognosis, which is regarded to be induced by intratumoral microbiota and inflammatory TME 139, 148, 149 (Table 2). It has also been found that abnormal serum levels of various amino acids in HNSCC patients, including increased citrulline and decreased arginine, are associated with tumor metabolism and can served as auxiliary diagnostic factors 150 (Table 2). These metabolic changes may be partially contributed by arginine metabolism of intratumoral microbiota.

Therefore, it is expected that the composition of intratumoral microbiota and its metabolic indicators will be incorporated into the biomarker spectrum, in order to improve the diagnostic accuracy and prognostic evaluation of tumors. It is possible to consider joint detection involving the gene abundance of Arg and ODC pathway in tumor and fecal microbiota, the systemic levels of arginine and ornithine, and the expression of Arg and ODC in tumor tissues, aiming to assess the activity of the microbiota-metabolism axis from multiple perspectives and infer the biological and immune status of the tumor (Table 2). Large-scale cohort studies and multi-omics analyses in the future will further clarify the clinical value of these indicators.

### 4.2. Therapeutic targets and intervention strategies

A thorough understanding of the intratumoral microbiota-arginine/ornithine metabolic interaction has provided us with numerous therapeutic targets and innovative ideas. The following are several potential intervention strategies:

#### 4.2.1. Metabolic enzyme targeted therapy

The inhibition and utilization of key enzymes such as Arg1/Arg2, ADI, and ODC is a direct intervention (Table 2). Clinically, PEGylated arginase (Peg-rhArg1, BCT-100) and ADI-PEG20 have been developed for arginine depletion in the body to treat tumors 28, 151, 152. BCT-100 converts arginine into ornithine, which may potentially promote polyamine synthesis with promoting tumor growth, while ADI-PEG20 converts arginine into citrulline, which cannot be utilized by ASS1-deficient tumors, thereby more effectively starving the tumors 28, 152. The irreversible inhibitor of ODC, difluoromethyl ornithine (DFMO), has been used clinically for the prevention of colorectal polyps and has shown tumor-suppressing and immune-active effects in neuroblastoma 153, 154 (Table 2). For the microbiota-related arginine metabolism, ADI inhibitors (L-Canavanine) are used to block the arginine metabolism of *Streptococcus*, and dose-dependently inhibit *S. anginosus* to convert arginine into ornithine 18. Therefore, drugs selective targeting microbial ADI/Arg may be developed to precisely regulate the arginine metabolism of intratumoral microbiota, avoiding the impact on host cells.

It is worth noting that combining metabolic enzyme inhibition with immunotherapy has great potential. Arg1 inhibitors (CB-1158) combined with anti-PD-1 antibodies were used in phase I clinical trials for solid tumor patients, in order to alleviate the arginine depletion mediated by intratumoral microbiota and MDSCs, with enhanced efficacy of immunotherapy. Its results primarily indicated good tolerances 155. In preclinical models, the combination of ODC inhibitors and ICB also showed a synergistic effect on reversing polyamine-mediated immunosuppression and enhancing T cell activity 25, 156, 157. Therefore, it is possible to develop novel strategies including the simultaneous inhibition of Arg1 and ADI to control the two pathways of arginine metabolism 158, 159. To sum up, metabolic enzyme targeted therapy provides a direct intervention for the intratumoral microbiota-metabolism axis, whose key lies in selecting appropriate patients, such as screening ASS1-deficient tumors for ADI therapy and controlling potential side effects.

#### 4.2.2. Microbiota editing and probiotic therapy

A strategy that directly regulates the composition and function of intratumoral microbiota can intervene the metabolic interaction from the source. Importing beneficial bacterial strains can reverse the unfavorable metabolic state of the TME. For instance, an engineered *E. coli Nissle 1917* has been constructed to reconvert tumor metabolic products into arginine in TME 98, 160 (Table 2). It significantly increases the local arginine concentration, enhances the tumor-infiltrating T cells and synergistically performs a stronger anti-tumor effect with PD-L1 antibody 98, 99. It suggests that altering microbial function via probiotics or synthetic biology can improve the metabolism and signal environment of TME. On the other hand, eliminating or suppressing tumor-promoting microbiota is also an important direction. The antibacterial peptides or phage preparations specifically targeting *F. nucleatum* could be used to reduce its products like polyamines and LPS, and improve the prognosis of CRC patients 161, 162 (Table 2). Currently, the phages for *F. nucleatum* revealed activity *in vitro* 136, 163, 164.

In addition, CRISPR-Cas delivery vectors are used to deliver gene editing systems that cause the loss of arginine/ADI functions into intratumoral microbiota. It can suppress specific metabolic functions without disrupting the microbiota ecology 165, 166 (Table 2). Although the microbiota editing technologies are still in the early stage, they represent the future perspectives of precise therapy in the tumor microecology. It should be noted that there are challenges including delivery and the survival of the strains, as intratumoral microbiota are usually deeply hid within solid tumors. Hence, it is necessary to develop targeted delivery systems for tumor tissues, while strictly controlling safety to avoid infection or systemic inflammatory responses.

#### 4.2.3. Optimization of traditional therapy for microbiota

The traditional therapy for microbiota involves antibiotics and dietary interventions. Systemic use of antibiotics in tumor treatment should be cautiously performed, as it may disrupt the beneficial microbiota and decrease the immune efficacy 167-169. However, local or short-term use of antibiotics may be beneficial in cases where certain bacteria promote tumor progress. The systemic antibiotic clearance of the microbiota presents more sensitivity to immunotherapy in pancreatic cancer mice 113, 167. Clinically, ciprofloxacin was attempted to add into chemotherapy for PDAC patients, and the results showed partly safety and improvement 167 (Table 2). Further developments may involve narrow-spectrum or targeted antibacterial strategies, using specific drugs to inhibit the microbiota with arginine metabolism while preserving the beneficial microbiota. This requires prior detection of intratumoral microbiota in patients and medication based on metabolic characteristics in order to achieve precise microbial treatment. Furthermore, a focus on diet, in particular high-fiber and low-red-meat diets will adjust the microbiota to produce more beneficial metabolites, such as increasing butyrate and reducing harmful amines, which may suppress tumor progression and improve immune function 72, 170, 171 (Table 2). Overall, the optimization of traditional therapy still requires more clinical validation, and it provides simple and feasible adjunctive therapy for patients.

#### 4.2.4. Combination with immunotherapy

Since the intratumoral microbiota-metabolism axis plays a key role in immune regulation, it is expected that it could be combined with immunotherapy to achieve synergistic effects 86, 172, 173. The metabolic enzyme inhibition and probiotics can alleviate the immune-suppressive state in TME, thereby enhancing the immunotherapy efficacy. For example, Arg1 inhibitors or polyamine blockers can inhibit the function of MDSC and Tregs, enhance T cell proliferation, and then administer PD-1/CTLA-4 antibodies to transform a "cold tumor" into a "hot tumor" 174 (Table 2). The combination of CB-1158 with PD-1 antibodies has been evaluated for safety and efficacy in clinical trials 155, 174, 175. Additionally, engineered bacteria can directly deliver immune-stimulating molecules. The genes encoding immune-active factors have been introduced into intratumoral microbiota through engineered bacteria, which can maintain continuous expression in the tumor site and enhance the immunotherapy efficacy 176, 177. Yoon *et al.* used attenuated *Salmonella* carrying the IFN-γ gene in mice, which significantly inhibited tumor growth 178 (Table 2). These strategies combined with metabolic regulation may simultaneously regulate metabolism and immunity to achieve more ideal anti-tumor effects. However, broad-spectrum antibiotics combined with ICB were found to weaken efficacy in some researches, suggesting the precise control of the intensity and timing of microbiota intervention should be explored 179. The metabolic and microbiota regulation should be selected based on different patients, and combined with immunotherapy or chemotherapy, to achieve individualized comprehensive treatment.

## 5. Discussion and Perspective

More and more pieces of evidence indicate that intratumoral microbiota profoundly affect the TME and disease progression by reprogramming the arginine-ornithine metabolic pathway. The intratumoral bacteria can consume and metabolize arginine, thereby altering arginine supplement and immune mediators in the TME (Figure 2). *S. anginosus* found in gastric cancer can increase ornithine accumulation through arginine metabolism and significantly inhibit the differentiation and infiltration of CD8+ T cells, then promote tumor proliferation and metastasis 18 (Figure 2). Similarly, the ability of intratumoral microbiota to decompose arginine is closely related to CRC progression. The microbial arginine depletion can increase ornithine content within tumor, thereby activating the polyamine synthesis mediated by NO synthase and ODC, which induce angiogenesis, polarization of immunosuppressive macrophage, and upregulation of Wnt/β-catenin signaling, ultimately accelerating CRC growth 26. Moreover, polyamines as crucial metabolites of the arginine-ornithine pathway, are termed as important mediators to promote tumor growth and immunosuppression (Figure 2). Tumor cells and myeloid suppressor cells perform excessive polyamine metabolism, and high levels of polyamines can cause immunosuppressive macrophage polarization, MDSC proliferation, and effective T cells suppression 51. This is typical in "cold tumors" like PDAC, where cancer cells to produce ornithine and synthesize polyamines, shaping a highly immunosuppressive TME 51, 180. Meanwhile, the by-products of arginine metabolism also synergistically promote tumor progression by reshaping the TME.

At the same time, the metabolites produced by intratumoral microbiota also regulate the host's amino acid metabolism and immune network through signaling pathways. SCFAs including butyric acid can act on the metabolic sensing pathway of T cells and inhibit histone deacetylase, thereby upregulating effector molecules such as IL-2 receptor (CD25) IFN-γ and TNF-α, enhancing the proliferation and anti-tumor activity of CTL and CAR-T cells 100 (Figure 3). Metabolites of tryptophan like indole derivative can activate the AHR signaling pathway and induce the expression of chemokines and cytokines like CXCL9 and IFN-γ, further promoting the proliferation of tumor-infiltrating T cells and inhibiting tumor growth 181, 182 (Figure 3). In digestive tract tumors, changes in microbial arginine metabolic pattern in CRC patients are related to the activation of the arginine and polyamine pathway in TME 26. *Pseudomonas* enriched in PDAC were found to be significantly associated with the abnormal enrichment of arginine and proline metabolic pathways 99. Therefore, the intratumoral microbiota-metabolism-immune interaction constitutes a crucial network that affects the occurrence and development of cancer for clarifying tumor mechanisms.

As the field advancing rapidly, it still faces many challenges and unsolved problems 183. Firstly, intratumoral microbiota presents a low biomass with the weak microbial signals in the samples that are susceptible to environmental and sequencing contamination, thus making it very difficult for it to be accurately identified and located 184. Secondly, the heterogeneity and dynamic changes of microbiota among different patients make it complicated to explore the mechanisms and clinical translation 2. Thirdly, many conclusions about the influence of the microbiota on host metabolism and immunity are mainly based on correlation analyses that need to be strictly proven. For instance, only certain microbiota were found to be associated with amino acid metabolism disorders in PDAC study, but the causal mechanism and specific mediators remain unclear, and require further experimental evidence to support 8. Additionally, our knowledge of how the intratumoral microbiota can reshape the immune function through metabolism is still limited, and more accurate analytical methods are needed. Nevertheless, deeper exploration for intratumoral microbiota is considered crucial for revealing processes of tumor immune evasion and metabolic re-programming, and contributing to developing new therapeutic strategies and biomarkers 11, 185.

It is expected that the research and applications targeting the intratumoral microbiota-metabolism-immune axis might progress to a breakthrough. The integration of advanced technologies such as spatial multi-omics would provide an entire picture of the interaction between intratumoral microbiota and host cell metabolism in *in situ* resolution, thereby precisely locating certain microbiota to affect the function of adjacent immune cells through which metabolic pathways 2. Said high-resolution analysis can uncover key bacterial-host metabolic interaction nodes, providing targets for intervention. Secondly, clarifying the metabolic characteristics of the patient's individual microbiota will promote the development of personalized intervention strategies. The individual intervention strategies could be formulated based on the spectral differences in the metabolites from patient microbiota, improving the treatment accuracy 11.

Therapeutically, combined immunotherapy strategies are to be focused on. Arg1/Arg2 inhibitors and ODC inhibitors combined with ICB can produce a synergistic effect, which can restore the L-arginine level and reduce immunosuppressive polyamines in the TME, thereby increase tumor-infiltrating lymphocytes and enhance ICB efficacy (Table 2). In mouse models, Arg1/Arg2 inhibitors could significantly increase the arginine and reduce polyamine, while increasing immune cell infiltration and activation, further significantly enhancing the efficacy of anti-PD-1 therapy 138. Moreover, engineered bacteria therapy also shows great potential. They can colonize in tumors and continuously produce beneficial metabolites or enzymes by modifying intestinal or intratumoral microbiota through synthetic biology, which can directly regulate the local immune response (Table 2). For example, *Nissle 1917* expressing IAA activated the AHR pathway and induced increased CXCL9 and IFN-γ, together with enhancing CD8+ T cell infiltration, thereby inhibiting tumor growth and improving survival 181, 182. This demonstrated that engineered bacteria-mediated metabolic intervention can trigger a persistent immune memory response to tumors. Finally, non-invasive biomarker monitoring based on the microbiota-metabolic axis is also an important direction. Changes in intratumoral microbiota and their metabolite spectra are expected to be useful for disease diagnosis and efficacy prediction (Table 2). The concentration of specific microbial metabolites in feces or blood and the composition characteristics of oral or fecal microbiota may become alternative indicators reflecting the immune metabolic state in TME 186, 187. The development of kits for these biomarkers will provide non-invasive and highly sensitive tools for cancer screening and assessing, which would be expected to be able to achieve early warnings and real-time evaluation of tumor progression and treatment response.

## 6. Conclusion

In conclusion, the research on intratumoral microbiota-arginine/ornithine metabolism-immune axis is revealing the complex and crucial regulatory levels within the TME. It integrates the tumor evolution in microbiology, metabolism and immunology, which not only deepen the understanding of the immunosuppressive mechanisms in the TME, but also provide novel perspectives for the development of anti-cancer strategies. With the application of multidisciplinary technologies and mechanism being studied, we might more effectively control intratumoral microbiota and its metabolites, further converting them into clinical advantages and establishing a new model for tumor diagnosis and treatment based on microbiota metabolic characteristics. In future, precise intervention of intra-tumoral microbiota-metabolism pathways and integration of immunotherapy approaches may generate a breakthrough for combating the tumor prognosis and improving treatment efficacy.

## Figures and Tables

**Table 1:** Summary of typical microbiota and their roles in arginine metabolism in various cancers.

Cancer Type	Typical Microbiota	Roles in Arginine Metabolism	References
Colorectal Cancer (CRC)	*Fusobacterium nucleatum*	Produces polyamines from arginine, activating Wnt/β-catenin pathway, promoting proliferation and metastasis.	19, 26, 136
	*Escherichia coli*	Arginine degradation via AST pathway, influencing tumor progression by enhancing angiogenesis and immunosuppressive macrophages.	26, 104, 136
Gastric Cancer (GC)	*Helicobacter pylori*	Arginine consumption via Arg pathway, depletes NO production, and induces immunosuppression.	25
	*Streptococcus anginosus*	Produces ornithine from arginine, promoting tumor cell proliferation and immune suppression.	137
Esophageal Cancer (ESCC)	*Fusobacterium nucleatum*	Converts arginine to polyamines, disrupting the polyamine metabolic balance and enhancing malignancy.	19
Pancreatic Cancer (PDAC)	*Pseudomonas*	Enriches with arginine and proline metabolic genes, promoting immune suppression via macrophages.	113
Bladder Cancer (BCa)	*Fusobacterium nucleatum*	Converts arginine to ornithine and polyamines, stimulating tumor proliferation and promoting immunosuppressive TME.	26
Melanoma	*Fusobacterium nucleatum*	Increases tumor progression through polyamine metabolism, modulating immune responses.	138
	*Porphyromonas gingivalis*	LPS production and immune modulation, contributing to tumor progression via NF-κB signaling.	99
Head and Neck Cancer (HNSCC)	*Streptococcus anginosus*	Increases Arg1 expression in macrophages, promoting immunosuppressive phenotypes.	139

**Table 2:** Summary of transformation targeting intratumoral microbiota-arginine/ornithine axis in TME.

Transformation	Therapy Directions	Targets	Mechanisms	Reference
Diagnosis	Microbiota diversity markers	α-diversity of intratumoral microbiota	Positively correlated with the prognosis of patients	143-145
		Abundance of specific microbiota (*Streptococcus, Fusobacterium*)	Enrichment indicates an upregulation of the arginine metabolic pathway, leading to immunosuppression and tumor progression	18
	Metabolic pathway markers	Genetic abundance of the bacterial Arg/ODC pathway & host expression of Arg1/ODC	By consuming arginine through ADI or Arg, ornithine or polyamines are produced, thereby altering the nutritional status of the TME	11, 146
		Serum arginine/ornithine/citrulline levels	Decreased arginine and increased citrulline indicate an imbalance in the metabolic pathway	150
	Non-invasive liquid biopsy	Detection of *F. nucleatum* in faeces	Associated with the risk of liver metastasis in CRC	104, 147
		Blood SCFAs/polyamine metabolite profile	Indirectly reflect the metabolic activity and immune status of the microbiota	139, 148, 149
Treatment	Metabolic enzyme targeted	Arg1/Arg2 inhibitor (CB-1158)	Relieve arginine depletion and restore T cell function	158, 159
		ADI-PEG20 (Arginine Exhaustion)	Selective starvation of ASS1-deficient tumors	152
		ODC inhibitor (DFMO)	Block polyamines synthesis and reverse immunosuppression	153, 154
	Microbial editing and engineered bacteria	Engineering *E. coli Nissle 1917* (arginine synthesis)	Supplementing arginine in the tumor tissue and enhancing T cell infiltration	98, 160
		Bacteriophage/antimicrobial peptide targeting *F. nucleatum*	Specific elimination of tumor-promoting bacteria reduces the production of polyamines	161, 162
		CRISPR-Cas editing of the microbiome genes	Weaken the specific metabolic functions of the microbiota without disrupting the ecosystem	165, 166
	Traditional therapy optimization	Narrow-spectrum antibiotics (ciprofloxacin)	Eliminate specific tumor-promoting microbiota and reinvigorate the response to immunotherapy	167
		High-fiber/low-red-meat dietary intervention	Adjust the metabolic profile of the microbiota and inhibit the polyamine pathway	170, 171
	Combined immunotherapy	Metabolic enzyme inhibitors + ICB (anti-PD-1/CTLA-4)	Metabolic intervention removes the immunosuppression of TME, and ICB activates T cells to kill the target cells	155, 174, 175
		Delivery of immune factors by engineered bacteria (IL-2, IFN-γ)	Continuously release cytokines, recruit and activate tumor-infiltrating lymphocytes	178

## Data Availability

The data presented in this study are available in article.

## Abbreviations

TME: Tumor microenvironment
Arg: Arginase
ADI: Arginine deiminase
Treg: Regulatory T cells
GPR43: G protein-coupled receptor 43
AHR: Aryl hydrocarbon receptors
ODC: Ornithine decarboxylase
SCFAs: Short chain fatty acids
OTC: Ornithine transcarbamylase
ASS1: Argininosuccinate synthetase
ASL: Argininosuccinatelyase
MDSCs: Myeloid-derived suppressor cells
iNOS: Inducible nitric oxide synthase
DCs: Dendritic cells
ESCC: Esophageal squamous cell carcinoma
IDO: Indoleamine 2,3-dioxygenase
GPCRs: G protein-coupled receptors
HDACi: Histone deacetylase inhibitor
I3A: Indole-3-carboxaldehyde
IPA: Indole-3-propionic acid
IAA: Indole-3-acetic acid
PDAC: Pancreatic ductal adenocarcinoma
CTL: Cytotoxic T lymphocyte
Th1: Helper T cell
CRC: Colorectal cancer
AST: Arginine succinyltransferase
HNSCC: Head and neck squamous cell carcinoma
DFMO: Difluoromethyl ornithine
LPS: Lipopolysaccharide
ICB: Immune checkpoint blockade
TAM: tumor-associated macrophages
